# Book Reviews

**Published:** 1822-11

**Authors:** 


### CRITICAL ANALYSIS

OF

### ENGLISH AND FOREIGN LITERATURE

RELATIVE TO THE VARIOUS BRANCHES OF

### Medical Science.

Quæ laudanda forent, et quæ culpanda, vicissim

Illa, prius, cretâ; mox hæc, carbone, *notamus*.—PERSIUS.

### DIVISION I.

### ENGLISH.

ART. I.—*Dublin Hospital Reports and Communications in Medicine and Surgery.* Vol. III. Hodges and M'Arthur, Dublin, 1822.

PART I.

1. *Report of the Whitworth Hospital, containing an Account of Dysentery, as it appeared in Dublin in the latter end of* 1818; *with a brief Account of that Disease as it appeared in Limerick in* 1821. By JOHN CHEYNE, M.D. F.R.S. ED. Physician General to his Majesty's Army in Ireland, &c. &c.

DYSENTERY has long been known in some parts of Ireland, ranking in frequency and fatality next to continued fever. During the seventeenth century it prevailed to a great extent, and proved destructive to the armies engaged in the civil wars. The disease was likewise common at different periods of the eighteenth century, particularly in 1740, when it followed an epidemic fever resembling that of 1818; the resemblance between the epidemies being rendered still more remarkable from the circumstance of dysentery having followed the fever in both instances.

During the rebellion, many regiments of raw and newly-embodied troops were sent into Ireland, particularly the Fencibles, from different parts of England and Scotland; and it appears, from the records of the Army Medical Board, that the soldiers suffered severely from dysentery,—particularly those quartered at Cork, Limerick, Waterford, Carrick on Suir, &c. The prevalence of dysentery and typhus fever at the period in question is to be, in a great measure, accounted for by the badness of the accommodation afforded to the army. The troops having been sent over for the purpose of putting down the rebellion, were, of course, disliked by those who favoured the insurgents, and not very welcome guests even to the well affected. The consequence was that, in the different towns in which they were quartered, the persons at whose houses they were billeted preferred giving them money to procure other lodgings, constituting what is called a *dry* billet. Refused admittance into the lodgings allotted to them by the public authorities, the soldiers were driven, of necessity, into the worst parts of the town, and in many instances were forced to take up their quarters in cellars under ground. In short, they generally occupied the lowest, the dampest, and consequently most unhealthy, situations that could be found.

Although this circumstance is not alluded to in the Report before us, we are satisfied of its accuracy, being related on the authority of a medical man who acted as surgeon to one of the Fencible regiments; and we deem it of some importance, as showing the cause why the disease was then so prevalent among the troops. Under the present improvements in the barrack and hospital regulations, we believe the remark of Dr. Poole and Dr. Brachen will be found generally correct,—that the inhabitants suffer in much greater proportion than the military. Indeed, abundant evidence appears of the dependence of epidemic dysentery on locality, and of its being endemic in certain low, moist situations, where it is fostered by penury and filth.

But to come to the Report more immediately under review.—In September, 1818, during the epidemic prevalence of fever in Dublin, dysentery became so prevalent as to render the fever-hospital insufficient for the accommodation of the patients; in consequence of which, wards were prepared for their reception in the Whitworth Hospital, which was placed under the superintendance of Dr. Cheyne, author of the present memoir. Every arrangement seems, on this occasion, to have been made which was calculated to carry into full effect the objects of the institution: indeed, it is impossible to praise in too high terms the judgment and *tact* displayed by military surgeons, in availing themselves of every means afforded by existing circumstances of providing for the accommodation and comfort of their patients; a skill acquired by constant exertion amid the scenes of carnage and disease, with which the late wars have rendered them but too familiar. It is well observed "that little benefit can be expected from mere prescription, unless an hospital be suited to the diseases which it contains,—unless proper regard be paid to comfort, in the supply of the patient's food, to the cleanliness of his person, to the spaciousness of the wards, to the purity of the air which he breathes, and to his feelings."

The following table will show the diet of the patients labouring under dysentery, as employed at the Whitworth Hospital in 1818-19:

| DIET. | BREAKFAST. | DINNER. | SUPPER. |
|---|---|---|---|
| Full. | White Bread, ½ lb.<br>New Milk, 1 pint.<br>or,<br>Rice, 4 oz.<br>New Milk, 1 pint.<br>Cinnamon and Sugar. | Mutton, ½ lb. raw, made into soup.<br>White bread, ½ lb. | White Bread, 4 oz.<br>New Milk, 1 pint.<br>or,<br>Flour, ½ pint.<br>New Milk, 1 pint.<br>Cinnamon, &c. |
| Middle. | As above. | White Bread, ½ lb.<br>New Milk, 1 pint.<br>Spice and Sugar. | Flour, ½ pint.<br>New Milk, 1 pint.<br>Spice, Sugar, &c. |
| Low. | Flour, ½ pint.<br>New Milk, 1 pint.<br>Spice and Sugar. | Rice, 2 oz.<br>New Milk, 1 pint.<br>Spice and Sugar. | Flummery, 1 pint.<br>New Milk, 1 pint. |

Sago, Indian Arrow-Root, Tapioca, Eggs, Tea, Chickens, &c. were occasionally given.
Rice-Water, Barley-Water, New Milk, *ad libitum.*

A distinct connexion was observed between the dysentery and continued fever,—the former frequently arising during the course of the latter, sometimes during convalescence, particularly at that period when there was the greatest liability to a relapse; sometimes dysentery ended in fever; sometimes fever in dysentery. The cases most severe in their symptoms, and most unmanageable in their treatment, were those arising in the course of fever; the mildest were those which had least connexion with it. Of ninety-eight cases of dysentery, thirty-three arose during convalescence from fever, fifteen during its progress, fifteen had their origin in exposure to cold and wet, and four arose from indigestion; the rest were doubtful.

Dysentery is one of those diseases, the history of which involves the doctrines of contagion; and probably there is no disease with regard to which more opposite opinions have been held on this point: even in the report before us, Mr. Dillon, surgeon to the Conmell Dispensary, states that the dysentery of 1818 "seemed to him contagious;" Dr. Poole is of opinion that "dysentery is contagious;" Dr. HALLARAN, that "it has been obviously contagious on many occasions." On the other hand, Dr. M. BARRY "does not consider the dysentery of Cork contagious;" and Dr. PERSTON, assistant surgeon to the 79th regiment, says "the disease had decidedly nothing contagious in its character." It may, perhaps, be objected, that Dr. Perston speaks of the disease as it prevailed at Limerick; but Drs. Barry and Hallaran are both practitioners in Cork. We know not how our readers might be inclined to decide between the learned Doctors, but we dare conjecture they will feel obliged to Dr. Cheyne, who has saved the necessity of a decision, and gets out of the dilemma by the ingenious contrivance of agreeing with both. "From these communications from Cork, I should be disposed to conclude that the inhabitants of that city are liable to dysentery in each of its principal varieties, both as connected with continued fever and as an endemic disease connected with intermittent fever; the former variety contagious, the latter not at all so." Such of our readers as did us the honour to peruse the account of the Barcelona fever in a late Number of this Journal, may naturally feel a little nervous at the prospect of our getting upon the inexhaustible subject of contagion a second time; but on this occasion we promise to be very moderate.

STOLL is very decided in his opinion that dysentery is not contagious: he allows, indeed, that the offensive exhalations of the stools may excite other diseases, but denies that they can specifically communicate the one in question; in proof of which he informs us, that he and his assistants were in the habit (and with impunity,) of snuffing up the exhalations from the stools of all the dysenteric patients every morning "totis naribus." That the disease is not contagious as it occurs in tropical climates, is the opinion of some of the most distinguished writers: it is sufficient at present to mention Mr. BAMPFIELD and the learned editor of the Medico-Chirurgical Review. The evacuations in this, as well as typhus fever, have been regarded as one of the principal means of infection; and much stress is laid on this point by most writers on Dysentery. That innumerable instances might be adduced similar to the fact cited from Stoll, is known to all; but the report before us furnishes us with a still more direct application of the virus, (if such it be,) as detailed in the following satisfactory, but very filthy, experiment:— Dr. Barry mentions that, in the years 1797, 1798, and 1799, dysentery prevailed to some extent among the soldiers of the Caithness Fencibles. "The surgeon, anxious to determine the question as to its infectious nature, caused the same glyster-pipe to be used, without cleansing, for those labouring under dysentery and those who were free from that disease: the latter, notwithstanding, were not infected; from which he concluded that the dysentery of Cork is not infectious."

On the other side of the question are to be found the names of some of the most distinguished practitioners of the times in which they lived,—PRINGLE, ZIMMERMANN, and CULLEN, and, of the present day, Messrs. DESGENETTES, COSTE, and PINEL. Among them the general opinion was, that the exhalations from the evacuations of dysenteric patients were the great source of propagating the disease; some of them likewise mention the actual contact of matters voided by stool. It is impossible to conceive any proof more directly contradictory of this latter doctrine than the experiment above quoted; and, considering the evidence on both sides, we are disposed to agree with those who regard dysentery as sometimes non-contagious, and at other times (under particular circumstances,) capable of spreading by infection. In short, we agree with Zimmerman, who says that the same dysentery is contagious or not, according to circumstances; which circumstances are principally found in crowded hospitals, where cleanliness is not sufficiently attended to. We believe the history of dysentery will show that it has seldom assumed an infectious character, until after it has prevailed for some time, and attacked a considerable number of persons; that it is not until the atmosphere has become charged with miasmata, emanating from the sick and their evacuations. The evidence of its infectious nature, from its spreading among camps and garrisons, is, for the most part, very doubtful; because the general privy becomes a focus of malaria, corrupting the surrounding atmosphere, and gives rise to the disease in the same manner as a piece of stagnant water will infect a whole town with intermittents. Indeed, there are many circumstances to show that the simple exhalation from putrid substances, without any thing specific in their nature, will cause deranged action in the bowels. M. VAIDY was sent to bury the bodies of about four hundred men and two hundred horses, which had been left on the field of battle for four days in the month of August, 1796, in the vicinity of Nuremberg. The heat had been great, and the stench was insupportably disgusting, particularly from the horses. The process of interment lasted several hours, during which time he became affected with nausea and cholic: on returning home, he was attacked with diarrhœa, ending in dysentery; and two, out of four, gendarmes, who had accompanied him, were similarly affected, and his horse died in the evening of the same day of cholic. Zimmerman mentions an instance of an individual who became affected with dysentery from smelling putrid blood, which had been kept in a bottle, by which means the putrid effluvia must have been concentrated. And to mention one more instance:—it rests on the authority of M. Desgenettes that he and several individuals became affected with bowel complaints, from the odour of a rotten hide.

These and similar instances render it obvious that such putrid emanations are capable of producing affections which sometimes end in dysentery, and which, therefore, render us rather sceptical as to the specific contagion of the disease. When a disease is really contagious, (as small-pox,) remove an individual labouring under it to a situation where all around are healthy,—they will become affected, and the disease be kept up: at least, this will happen in the great majority of cases. It holds good, for instance, with regard to small-pox, which has never been banished from London, notwithstanding the assistance we have received from vaccination. But, with respect to dysentery, this does not seem to be the case: indeed, there are not wanting instances of the converse,—viz. placing healthy individuals in the same houses with the infected, and exposing them with impunity to all the force of the contagion. Dysentery prevailed epidemically, in 1808, in Jutland and Holstein, and proved extremely fatal. At Horsens, a village in Jutland, there was stationed a garrison of French troops, amounting to four hundred men, who, as well as a like number of Spaniards, were all quartered with the inhabitants, among whom the disease was making great ravages; yet only two of the soldiers became dysenteric. The troops had recently arrived in the country, and their escape is easily accounted for by supposing the disease epidemic, and that they had not been long enough exposed to the causes in which it originated. But, if we allow the disease to have been contagious, how then is the circumstance to be explained?

Dr. Barry states that he has frequently seen dysentery at Cork, "among the inhabitants of a low confined district;" and that "he has treated single patients, from whom he never saw it spread." And, to conclude this subject, it appears, from the testimony of Pringle and Zimmerman, (both contagionists,) that the simple removal of a camp to a different situation is capable of diminishing, or entirely removing, the prevalence of the complaint.

The symptoms of dysentery, as described in the work before us, were a remarkably bad taste in the mouth, which the author is of opinion often exists when there is a deficiency of bile in the intestines; considerable flatulence, followed by rigors; griping pains, frequent inclination to go to stool, with continued unsatisfactory efforts to evacuate the contents of the bowels. The excretions were loose, generally mucous, and, in the majority of cases, mixed with blood on the very first day of the attack. It is remarkable that scybala were not observed in any instance, or in any stage of the complaint; but the evacuations presented the other varieties characteristic of dysentery, consisting of different forms of feculent matter, alone, or mixed with mucus, blood, or pus; of mucus, blood, or pus, alone and unmixed; of these substances variously combined; and, when the disease was near a close in some instances, portions of coagulable lymph, or shreds of membrane, were detected. The functions of the skin and liver were remarkably disordered throughout: the former was dry and hot, and the secretion of bile seemed often to be suspended for several days.

The cases differed very much from each other in point of severity; the symptoms which guided the prognosis being the tenderness to pressure, the frequency of the stools, and the degree of fever, especially as regarding the quickness of the pulse. After the disease had run its course unrelieved for twelve days or a fortnight, emaciation generally took place, which, added to considerable urgency of the symptoms just mentioned, was regarded as extremely unfavourable. The last stage was marked by great wasting of the flesh, supine posture, and involuntary stools; "a thin reddish secretion flowing without check, sordes on the teeth, hiccup, tendency to delirium, difficulty of swallowing, and a pulse like a thread." The disease proved fatal in the majority of cases in which it had existed more than a week prior to their admission into the hospital, and scarcely one individual recovered where emaciation had taken place. It was difficult or impossible, in the early stages, to ascertain whether ulcerations of the mucous membrane of the intestines had occurred or not; but, in the more advanced stage, this could sometimes be ascertained from the appearance of the stools.

When the disease passed into the chronic form, the patients became affected in its progress with various modifications of dropsy, particularly ascites and anasarca; and several examples occurred of an effusion into the cellular texture of the lower extremities, resembling phlegmasia dolens: this appearance was observed in both sexes.

Death seemed in some instances to originate in a translation of the disease to the lungs, giving rise to oppressed respiration, with cough and pain in the chest. Dissection, in every instance, showed the existence of accumulation of blood in the substance of the lungs, and effusions into the cavity of the pleura or into the bronchia. Another cause of sudden death, in some instances, was the escape of the contents of the bowels into the sac of the peritoneum, from the ulceration having penetrated their coats.

Death generally occurred either in the commencement of the attack, or not till it had existed for a considerable period, so as to give the diseased actions time to run their course. On this account no opportunity was offered of ascertaining, with any satisfactory precision, the symptoms which denoted the commencement of ulceration, as this could only be done by examinations during all the different stages of the disease.

The appearances which were found in the various dissections are thus described:

"The mucous membrane of the stomach and small intestines sometimes presented an inflamed appearance, which in general became more remarkable as we approached to the great intestines; then ulceration began to show itself, at first superficial, afterwards laying bare the muscular fibres of the intestines; the ulcerations became larger, more numerous, and deep, as the rectum was approached; but it was remarked that the last three or four inches of the rectum were sometimes pretty sound. The peritoneum was found less diseased than might have been expected.

"The deaths which took place when dysentery was in its first stage, were owing to fever or to some other fatal disease which concurred. When owing to fever, a peculiar state of the mucous membrane of the stomach and intestines was noted. This membrane was found of a more or less deep red or purple colour; it was rather thickened, soft, and pulpy; the least violence destroyed the continuity of the surface, which had lost its healthy smoothness, was uneven, and not unfrequently rough and granulated, the blood-vessels being enlarged and loaded with blood: where the alteration in texture was inconsiderable, there were numerous spots of a deep red colour, which at first resembled extravasations, but, on close examination, seemed to be blood-vessels, ramifying in an arborescent form, and connected with veins. The stomach contained a viscid mucus, firmly adhering to its coats, and mixed with an opake yellow or whitish matter; the contents of the large intestines were fluid, and of a yellowish green colour."

Besides these general phenomena, some individual peculiarities are worthy of note. A woman, who laboured under pulmonary disease with much fever, was attacked with pain in the abdomen, tormina, and discharges of blood by stool. The mucous membrane of the intestinal canal was highly vascular throughout its whole extent, but without any degree of abrasion; "proving that a free discharge of blood may from this, as from other mucous surfaces, take place without previous ulceration or erosion."

Mary Tully was taken ill on the 17th of September, and died on the 22d, having suffered much from pain in the abdomen, with excessive tenderness to pressure; the disease ran its course unchecked by the remedies employed. In this case the mucous lining of the stomach and intestines was "in very many places elevated by air, which was interposed between it and the subjacent coat." There were numerous bright red spots, some of which contained air-bubbles; the vessels of the membrane between these florid portions were impervious to minute injection. The ileum was free from appearances of disease; but the internal surface of the rectum and sigmoid flexure of the colon were red with some " black rough stripes" and a few minute ulcers.

The case of Elizabeth Curran is one which likewise merits attention. This patient, after exposure to cold, was attacked with rigors, pain in the abdomen, tormina, tenesmus, and bloody stools. About three weeks after the commencement of her illness, she was admitted into the hospital, (November 22,) when there was swelling of the belly, particularly about the umbilicus; the abdomen at this time was soft, but very tender to pressure. These symptoms resisted the use of purgatives, baths, calomel, opium, and bleeding; and, by the 7th of December, the tumefaction had increased so much as to give her the appearance of one in the advanced stage of pregnancy. An enema having been ordered, it was found impossible to administer it, and, on examination, a stricture was now discovered, about four inches up the rectum: a flexible catheter was then carried beyond the obstruction, and through it the enema was injected. On the 10th, *many quarts* of tepid water were thrown up: these were subsequently returned, mixed with a considerable quantity of excrementitious matter, and the swelling partially subsided, but without any relief. Opium alone seemed to have any effect in alleviating her sufferings, which were terminated on the 13th by death. " On dissection, the intestines were found greatly distended, the small intestines being *seven*, and the great intestines *nine*, inches in circumference; at four inches from the anus there was an extensive hard stricture, through which the finger could not be passed; the coats of the intestines were nearly an inch in thickness, which was chiefly owing to a dense white fibrous matter interposed between the peritoneal and muscular coat; the mucous membrane, both at and above the stricture, was in a state of ulceration."

An appearance is described, which it is important for those who conduct morbid dissections to have in view. There were, in several instances, about the rectum and colon, numerous regularly round holes, large enough to admit the head of a pin, which were at first mistaken for ulcers; but more extensive examination led to the opinion that they were the enlarged mouths of mucous ducts, and which frequently contained a gelatinous secretion. Were it not rather foreign to the present analysis, we could point out many instances in which similar cautions are necessary, having frequently had occasion to witness dissections in which appearances were said to be the effects of diseased action, particularly of inflammation, where, in reality, they had resulted from transudation and other changes occurring subsequent to death. With regard to the possibility there is of mistaking the open mouth of the duct of a mucous gland for an ulcer, we beg to refer our readers to some interesting remarks by Mr. SHAW, contained in this Journal, (vol. xlviii. p. 187,) from which it appears that this may occur in the living subject. Our own experience agrees with that of Mr. Shaw, for we have frequently seen the tonsils after quinsy, with an apthous state of the fauces, assume, and retain for a considerable period, the appearance of being studded with little white ulcers: but, on removing this supposed slough with the point of a probe, no ulceration is to be found beneath it, but a clean follicle in which this white secretion had been lodged. In case any of our readers should think this condition of the tonsil too common or too obvious to require or justify these observations, we beg to add, that we have ourselves known a case of this kind kept under treatment for three months.

We now come to the part of this report which is of most importance in the history of every disease—the method of cure. Nothing can be more absurd than the system of laying down certain fixed rules for the treatment of different complaints, regarding rather their place and name in the nosological scheme of the day, than the various modifications of symptoms, which are always to be found by those who study disease at the bedside of the patient. A very general rule, for instance, with regard to dysentery, is to give purgatives till the scybala be expelled, and the load supposed to result from their presence be removed; a direction which it would have been somewhat difficult to follow in the present instance, where extensive and careful examination showed that no scybala existed; and purgatives, instead of giving relief, in many instances greatly augmented all the sufferings of the patients. This absence of scybala, although they are so much insisted upon by CULLEN and others, has frequently been remarked by writers of the first repute. Among others, Sir JAMES M'GRIGOR states (see Medical and Surgical Journal, No. iii.) that he does not think six out of five hundred dysenteric patients passed scybala; and Mr. BAMPFIELD thus expresses himself, "In India, and within the tropics, I have very rarely observed the scybala, which I was particularly led to expect in the commencement of dysentery; and, with the exception of two instances, I have very generally found that the fæces which were evacuated, whether produced by the action of cathartic medicines or not, were what are commonly denominated loose excrements."

In the mild forms of dysentery,—that is, where it was unattended with much pain or fever, the exhibition of some gentle laxative, followed up by repeated doses of Dover's powder and low diet, were sufficient to remove the symptoms. In the more severe, where there existed considerable fever, tenderness of the abdomen, and mucous stools tinged with blood, venesection was had recourse to, with neutral salts, warm baths, and opiate diaphoretics. The practice of blood-letting, though forbidden in many books, is clearly borne out by analogy, and in the present instance justified alike by the symptoms during life and appearances after death. Besides the relief afforded to the pain and tenderness on pressure by blood-letting, it likewise had the effect, not unfrequently, of immediately improving the character of the evacuations, when purgatives had entirely failed to do so. "Those who object to venesection in this form of dysentery, have surely never witnessed the great relief which profuse hemorrhage from the bowels sometimes affords to the patient in dysentery." The use of the lancet was not confined to the early stages of the disease; it was frequently employed, with seeming advantage, even when there was ground for supposing that ulceration had already taken place in the mucous membrane. In agreeing with Dr. Cheyne as to the propriety of general depletion, where unequivocal symptoms of inflammation exist, we must at the same time remark, that we think local bleeding does not seem to have received the attention it appears to us to deserve. It was only "when, in the advanced stage of the disease, there was much tenderness or pain confined to one region, that leeches were preferred to the lancet;" and we are cautioned against the risk of patients dying from the effusion of blood after the application of leeches, as the author has known patients sink from this cause, who would have borne a small bleeding from the arm. Now admitting, as we freely do, the propriety of the caution, we cannot help suspecting that the instances to which he alludes, (of what is certainly a very rare occurrence,) have made rather more impression upon the author than they ought to have done. With regard to children, it is a very necessary circumstance to keep in view, the vascular state of the cutis in young subjects often giving rise to profuse, and sometimes fatal, bleeding from the leech-bites: but, with respect to adults, this, in all ordinary cases, would be a chimerical source of apprehension.

In the cases requiring depletion, a preference was given to the saline laxatives,—such as sulphate of magnesia or the tartrate of soda and potass, with a very minute proportion of the tartarized antimony. With respect to ipecacuanha, no very decided opinion is expressed; at which we feel rather disappointed, regarding that drug with considerable partiality; but castor-oil incurs the censure of frequently producing "much aggravation of tormina and tenesmus," and hence requiring the combination of an opiate. This effect of castor oil we have ourselves frequently witnessed, and do not regard it as properly ranked among our *mildest* purgatives. One of the laxatives very highly recommended to Dr. Cheyne was the supertartrate of potass: it was the protegee of a physician, who regarded this medicine as possessing extraordinary antiseptic powers, and who "conceived that dysentery essentially depended on the active operation of a prevailing septic principle in the body." The salt was to be very finely levigated, and the dose was half an ounce every fourth or sixth hour. Some patients treated in this manner recovered, who, in Dr. Cheyne's opinion, would have sunk under any of the methods of cure then in use, and the cream of tartar became a favourite in the hospital during the continuance of dysentery: yet in many instances it failed. Our author is of opinion that it excels most other purgatives in the property of "bringing down the bile," and endeavours to explain in this manner its favourable operation, having frequently seen much relief follow the evacuation of bile in this complaint. We are not sufficiently convinced of its superiority to other remedies of a similar kind to feel much concern about its *modus operandi.*

Various forms of glyster are mentioned as useful, particularly those containing opium, equal parts of lime-water and milk, or a solution of the superacetate of lead; others of a stimulating nature were likewise tried,—such as the common black wash, a weak solution of the argentum nitratum and oil of turpentine, rubbed up with mucilage. These had no effect in checking the disease; but, on the other hand, did not excite so much irritation as might naturally have been looked for.

The opinion formed by the author of the effects of opium and mercury are contained in the following passage:

"But there were cases requiring a procedure still more decided; cases wherein the disease had resisted the foregoing method so long as to excite an apprehension of extensive ulceration, or in which it began with unusual violence, or in which, after proceeding moderately for a time, symptoms of great danger suddenly arose,—as, for instance, intolerance of the slightest pressure, agonizing pain, unceasing tenesmus, great pyrexia: in these cases, the method recommended by some of the late writers on Tropical Diseases was used with advantage. In the course of a single hour the patient has been largely blooded, has taken two grains of opium and a scruple of calomel, has had the warm bath, and been swathed in flannel, by which means we have obtained breathing time to pursue our plans more deliberately. Were the same cases again to be placed under my care, I would not hesitate to prescribe opium in doses of four or five grains, as it was the opium chiefly which seemed to me to arrest the progress of the inflammation, and whatever in such a case procured respite for the patient from agony sometimes proved of permanent benefit. After the large dose of calomel and opium, calomel in doses of five grains, with half a grain of opium, every third or fourth hour, was given till it produced ptyalism: as soon as the mercurial influence was apparent from the state of the mouth, the mercurial was laid aside, and a mixture with balsam of capivi was given every fourth or sixth hour, from which the greatest relief was often obtained: the fæces, from being of a bottle-green, and mixed with mucus (the effect of the calomel and opium,) and passed with tenesmus, became more natural in appearance, and were voided less frequently; the patient considered himself cured, and in many cases his recovery followed, although not without interruptions, requiring various changes in the form of the anodyne. Such changes, it may be observed, were generally productive of benefit: thus, the chalk julep, or catechu mixture, with laudanum, when the capivi draught lost its effect, were substituted. It results from a consideration of the cases in my possession, that venesection, calomel, and opium, followed by the capivi mixture, with farinaceous diet, proved more successful than any other method which was adopted in the severest cases; yet it often lamentably failed, of which sufficient evidence will be found in the table of unsuccessful cases. It ought not to be forgotten that, when the skin was restored to a natural state by the mercury, the case generally proceeded favourably."

If such beneficial effects result from the use of mercury, it may excite surprise that it was not more generally employed; but the praise contained in the passage just quoted must be received with considerable limitation, for the remedy occasionally did harm: in some instances refusing to affect the mouth, and in others not relieving those who were salivated, even in the early stage of the complaint. In the more advanced state, where ulceration was supposed to have occurred, or where emaciation had taken place, mercury was useless, if not injurious. This position seems at variance with that of Dr. James Johnson. "I have only to add," says he, "that, since my return to Europe, I have never met with a case of dysentery, when I had the treatment from the beginning in my own hands, that did not give way to mercury and its auxiliaries, as before directed, and generally with more facility than between the tropics. In many cases of chronic dysentery, too, which I have met with among French prisoners and others, the practice (with some slight modification, principally in the *quantity* of the chief remedy,) has succeeded beyond my expectation, *where the degree of emaciation and the extent of local derangement* had rendered the prospect of a cure almost hopeless."*

In order to illustrate the different methods of treatment set forth in this essay, and the morbid phenomena resulting in the intestinal canal from the disease, thirty cases are subjoined; but as all these, without exception, proved fatal, we have no means to judge of the comparative merits of various remedies. Why does not the author give some of his successful cases?

* *The Influence of Tropical Climates, &c. &c.* By James Johnson, M.D. p. 208.

These details occupy twenty-three pages, but our limits forbid us to enter into particulars: indeed, we have already alluded to the individual cases presenting peculiarities worthy of note.

To the Report of Dr. Cheyne is attached a supplement, including an account of dysentery as it appeared in the 79th regiment at Limerick, in 1821: this account is preceded by the history of seven cases which occurred in the same family, and which would appear to afford an example of the disease being infectious.

The statement of Dr. Perston, assistant surgeon to the regiment, affords strong proof of the dysentery having originated in exposure to putrid effluvia, particularly during the night; the first cases, among the inhabitants of the town, having originated in the vicinity of extensive slaughter-houses, soon after an "immensity of offal" had been left to rot in the open air. Of the troops, several were seized when on guard during the night at the gaol, in the vicinity of which there is a great accumulation of all sorts of "dirt and filth." Other sources of noxious exhalations presented themselves in the confined and narrow streets, where cleanliness is little attended to, and in the bed of the Shannon, which had receded considerably from its banks during the long-continued droughts. The gentleman by whom the account (a clear and distinct one,) is given, thinks decidedly that the disease was not contagious.

The history of the symptoms resembles that already detailed: in the treatment, however, bleeding seems to have been practised more vigorously; "from thirty to forty, or more, ounces of blood were constantly taken from the arm." This was frequently followed by permanent relief; but, when the urgent symptoms returned, general bleeding was again had recourse to, and repeated as often as circumstances seemed to demand. The reader must keep in mind that practice of this kind, however successful among strong men, well fed and well clothed, cannot with safety be followed among the debilitated inhabitants of crowded cities.

Dr. Perston employed mercurial frictions on the region of the liver to a considerable extent, having observed that viscus to be always deeply involved in the disease, generally enlarged,—sometimes with entire disorganization of its structure.

2. *A Report of Cases of Aneurism, in which Operations were performed in the Richmond Surgical Hospital, Dublin.* By C. TODD, Esq.

In the two first of these cases, the external iliac was tied in the manner recommended by Sir ASTLEY COOPER; and in one of them the vessel was subsequently taken up a second time, nearer to the heart, according to the plan recommended by Mr. ABERNETHY. Both patients died, but from causes which cannot fairly be regarded as connected with the operations. The third case is that of a farmer, thirty years of age, who, after a day of hard labour, found a pulsatory tumor in the ham rather larger than an egg. This was judged a fair instance for attempting a cure without operating, and compression was applied to the artery in the groin, by means of an instrument resembling a truss, contrived so as to exert pressure only on one point, leaving the circulation by the anastomosing branches, free. Although the tumor obviously diminished, yet the patient could not be persuaded of the utility of the plan adopted; in consequence of which, and his suffering from confinement and low diet, the operation was performed. Two ligatures were applied; one as high as the vessel was separated from its connexions, the other as low. On the fourth day, (September 4,) he had slept but little during the night; had insatiable thirst and oppression in the chest, with a frequent hard pulse, and some erysipelatous inflammation about the wound. He was bled to the extent of eight ounces, and purged; but the symptoms, on the 6th, had become considerably aggravated, with broken sleep, agitation, anxiety, and violent palpitations at the heart. Pulse hard, and very frequent. He was now bled again, and took digitalis, by a repetition of which means he gradually recovered. By the 11th the digitalis was omitted; and on the 19th and 20th the two ligatures came away; after this the patient suffered from some fistulous openings in the thigh, but ultimately got well. The unfavourable symptoms are attributed to inflammation running along the inner membrane of the artery to the heart itself; but there is not sufficient evidence to determine this point in a satisfactory manner.

The fourth case is interesting, from the same attempt having been made as described in the preceding to effect a cure, by forcing the blood into the anastomosing branches. The aneurism, which was situated in the ham, arose from a strain caused by a stone slipping from under the heel, and about a week after the accident had attained the size of a walnut. In addition to the pressure made upon the artery in the groin, the patient was copiously bled; but no diminution seems to have been effected, and, at the end of about four months, the tumor assuming a conical form, and being evidently on the increase, it was deemed necessary to operate. Only one ligature was applied, which came away on the nineteenth day, and the patient did well.

These cases, it is to be observed, are related principally with a view of recommending this preparatory process before the operation is performed. The author thinks that many cases of mortification might have been prevented by such delay as would have allowed the anastomosing vessels to have made some progress in their new office; and he is very sanguine in his expectation that this desirable end may be facilitated by compressing the principal artery of the limb for a few hours every day, the period of its continuance varying under different circumstances. Indeed, Mr. Todd seems not without hope that such means may lead to complete recovery, without the necessity of operating at all: this idea is founded on the fact of aneurism sometimes undergoing a spontaneous cure, and that the means he has recommended have a tendency to assist nature in her salutary efforts. The subject is certainly deserving of attention, but at the same time it must be acknowledged the cases in question hardly afford much inducement for favourable anticipation: indeed, some may be inclined to suspect that the irritation kept up in the artery by the pressure of the instrument might have tended to produce those bad effects in the third case, which the author himself attributed to inflammation spreading along the inner membrane of the artery. Another objection to this method is, that even if it succeed in arresting the progress of the aneurism, still there is the risk of an operation becoming necessary at some period, however distant. It is true there are examples of permanent cure effected by this method of compression; one of these is mentioned by Richerand, and several are recorded by M. Dubois and others; but, for the reasons assigned above, we believe it will scarcely be regarded in any other light than as preparatory to a more radical cure by ligature.

3. *Report on Puerperal Fever.* By John C. Douglas, M.D.

This report consists of answers to certain queries contained in a circular letter from the Board of Health, in 1820, a form of composition rather tedious to the general reader: indeed, it is obvious that, to publish these queries at full length, is a work of supererogation, as a perusal of the answer must naturally suggest the question that gave rise to it. On this account, we shall take the liberty of omitting these altogether, and give only the remarks of Dr. Douglas, who appears well acquainted with the subject of which he treats.

With a view of reconciling the differences of opinion that exist with regard to the nature of puerperal fever, Dr. Douglas proposes to divide the disease into three species, which he places under the following heads,—viz. Synochal puerperal fever; Gastro-bilious puerperal fever; and Epidemic, or Contagious, puerperal fever. The name of the first is expressive of its nature, which he regards as purely inflammatory, resulting from exposure to cold, or some other accidental cause wholly unconnected with contagion. Blood-letting freely employed, and repeated as the symptoms may indicate, is the chief remedy recommended in this variety; antimonials, purgatives, enemata, and fomentations, being the requisite auxiliaries. We are cautioned that the pulse is frequently deceitful, and the pain is therefore held to be a surer criterion of the inflammatory action: the same remark applies to all the forms of abdominal inflammation. The distrust with which venesection has frequently been regarded in this and similar ailments, he regards as resulting from the inefficient manner in which it is had recourse to by timid practitioners.

In the *gastro-bilious puerperal fever*, the inflammatory character is not so quickly assumed nor so manifestly developed; yet it does exist, and advances progressively, although more obscurely. The pulse not "bounding and incompressible," as in the former case, but "frequent, hard, and concentrated." Blood-letting here, too, must be employed, but more sparingly; and it is said to be of "paramount importance" to excite the action of the mucous membrane of the bowels in the early stage of the disease. With this view, ten or twelve grains of calomel are to be given immediately after the phlebotomy, and followed up with an ounce of castor-oil, combined with other active purgatives. By this means the progress of the diseased actions is frequently arrested, "particularly whilst the morbid action of the peritoneal covering is under a kind of panic from blood-letting, and before that inflammatory action can re-organize its broken force." The causes of this are similar to those of the former variety, acting upon constitutions predisposed by debility and derangement of the digestive system.

The third form of the disease, it will be observed, is the one more peculiarly known by the name of puerperal fever, agreeing with the two former varieties in the existence of abdominal inflammation, but differing essentially in the detail of symptoms. We give the author's description, as follows:

"The sensorium here is seldom in any degree disturbed; whereas in the others it is so frequently, and even sometimes is excited to high delirium. The pulse here is usually, from the moment of attack, soft, weak, and yielding, and in quickness often exceeds 160; whereas in the first species it is full, bounding, and incompressible; and in the second small, hard, and concentrated, and in both moderately quick. The eye, instead of being suffused with a reddish or yellow tint, as in the others, is here generally pellucid, with dilated pupil. The countenance, instead of being flushed as in the others, is here pale and shrunk, with an indescribable expression of anxiety; an expression altogether so peculiar, that the disease could, on many occasions, be pronounced or inferred from the countenance alone. The surface of the body, instead of being, as in the others, dry and of high pyrexial heat, is here usually soft and clammy, and of heat not above the natural temperature; and not only is the skin cool with clammy exudation, but the muscles, to the impression of the finger, feel soft and flaccid, as if deprived of their

*vis insita* by the influence of the contagion. Indeed, there is such prostration of muscular strength and depression of vital principle, from the very onset of the attack, that I must suppose the contagion to act upon the human frame, probably through the medium of the nervous system, in a manner analogous to that of the contagion of the plague; and perhaps the African plague does not commit greater havoc among an equal number of infected persons than puerperal fever in this country; nor is puerperal fever less quickly fatal than the plague itself."

The treatment recommended is to begin with ten grains of calomel combined with two of opium, and the exhibition of a purgative enema; after the operation of this, from two to four dozen of leeches are to be applied to the abdomen, and the bleeding encouraged by the application of flannels wrung from warm water. Three or four hours after the calomel and opium, three drachms of pure oil of turpentine are to be administered in a little syrup and water; and this draught is to be followed, at the interval of an hour, by an ounce of castor-oil or some other brisk purge. Other remedies may be given, according to circumstances, but Dr. D. "would not be disposed to repeat the internal use of turpentine oftener than twice in any case whatever." By *repeating* it twice, does the author mean that he would give it three times? When the debility is great, the abstraction of blood may be altogether omitted, and a flannel dipped in turpentine applied to the abdomen for a quarter of an hour. Respecting this latter, the author states that he considers it, "*when judiciously administered*, more generally suitable, and more effectually remedial, than any medicine yet proposed." It is nearly seven years since the author first published an equally favourable report of this remedy, during which period more extended observation has not given him cause to alter his opinion: on the contrary, he is convinced that he has seen individuals recover under its use, after every hope from the usual methods of cure had been relinquished.

With regard to the comparative degrees of mortality, Dr. D. calculates that the fatal cases of sporadic fever scarcely rate so high as one in six; whereas not above one half of those attacked with the truly epidemic form of the disease recover. He therefore regards the latter as a very aggravated modification, if not essentially different from the former. Few seasons have passed without his having occasion to prescribe for cases of the sporadic form in private practice, but the truly epidemic puerperal fever seems confined to the Lying-in Hospital. In the year 1803, the disease was epidemic; in 1809, 10, and 11, it became so again; and a third time in 1819-20. When the puerperal fever is truly epidemic, he regards it as contagious, generally speaking, only to lying-in women; but that instances have occurred of delicate females, under other circumstances, being attacked with the disease from much exposure to the contagion; and that such contagion may be carried into private practice by those much engaged in hospital duties during the prevalence of an epidemy. The contagious puerperal fever of Dublin is, in his opinion, "neither more or less than a malignant fever of a typhoid character, accompanied with an erysipelatous inflammation of the peritoneal covering of the stomach, intestines, and other abdominal viscera." The answers to the queries regarding its history and the circumstances of its prevalence are connected with the opinion just quoted, which differs little from that of some others, and which, if correct, may serve to account for its frequent occurrence in large towns and crowded hospitals.

[To be continued.]

---

### DIVISION II.

### FOREIGN.

ART. II.—*Médecine Pratique éclairée par l'Anatomie et la Physiologie Pathologiques.* Par J. CRUVEILHIER, D.M. &c. &c. 8vo. pp. 183.

IT is justly observed, by the author of the work before us, that the tendency to generalize, so natural to man, is nevertheless by no means suited to the character of the human intellect, which is obliged to creep slowly from facts to facts before it can attain to the discovery of important truths. This remark might be amply illustrated by the history of medicine; for, perhaps, no other branch of study has suffered more from the love of theory, and from the hasty formation of unsupported systems. But, after the fall of many an airy fabric, men have at last awoke from the species of castle-building in which they had so long indulged. Times are changed since SYDENHAM ventured to despise the publication of particular cases; for Sydenham, though a practical physician, was theoretical enough to fancy that a method of curing diseases might be laid down, for which we have not yet half the materials. It is well, therefore, that diligence has at length been diverted from the construction of systems to the more necessary task of accumulating facts.

Engaging as a labourer in this ample field, M. Cruveilhier has determined to publish from time to time the cases of chief importance furnished by his practice. "Let clinical observation (he says,) determine our treatment and remedies, and then it will be in vain to appeal to theory." The views, therefore, of our author are *practical;* and, as he has omitted no opportunity of instituting examinations after death, the name of his work is not altogether inappropriate; although a title so general as

"Practical Medicine illustrated by Pathological Anatomy and Physiology" might lead one to expect a voluminous system already completed, instead of a single volume containing cases illustrative of a few particular diseases.

The principal subject of the present volume is a disease of the stomach and intestines of infants, which does not appear to have been as yet sufficiently described. Its symptoms are analogous to those of acute hydrocephalus: so also is the morbid alteration of structure to which it leads; for, as the latter is attended by a softening of the substance of the brain, this disease produces a gelatinous disorganization of the splenic extremity of the stomach, without inflammation, suppuration, or gangrene, but ending often in spontaneous perforation. The intestines are liable to the same alteration. Nor is the disease entirely confined to infancy, but sometimes occurs in adults, at the close of long and acute disorders. The treatment of it in infants, the author complains, is frequently erroneous, through an indolent and absurd practice of attributing all the symptoms to dentition or to worms.

This, the principal treatise, is preceded by shorter essays on Croup and Hydrocephalus; and, to complete the volume, the author relates his attempts to determine the seat of pulmonary tubercles, describes a new bandage for fractures of the clavicle, and announces the discovery of a new vegetable febrifuge remedy.

We proceed to give a brief analysis of the articles above enumerated. That upon Croup possesses no claim to novelty: the disorder is stated to consist in an inflammation of the mucous membrane of the larynx, trachea, and bronchial tubes, in consequence of which a false membrane is thrown out. It is said to differ from other inflammations only in the importance of the passages which it obstructs. Its sudden and insidious attacks are well described. In the early stage, the author considers that its course may *always* be arrested; and the treatment which he recommends consists,—1, of copious bleeding, local and general; 2, of emetics and cathartics, especially calomel and glysters; 3, blisters on the throat are highly praised; and 4, as a last resource, tracheotomy is advocated. The greatest vigilance is properly inculcated in guarding against a recurrence of the malady.

In the second article, upon Hydrocephalus, M. Cruveilhier proposes to resolve three important questions, which he thinks have been left undetermined by others.

1. What are the characteristic symptoms of cerebral disorder from its commencement?

2. What is its seats, and what the organic lesion which constitutes it?

3. What is the most appropriate treatment?

The discussion to which these questions lead is conspicuous for good arrangement and for a faithful description of symptoms; but, with the exception of a part of the treatment, we are not aware that any thing is adduced which was not generally known before.

The following symptoms, it is stated, may always be depended upon:

1. Irregular respiration; sometimes slow, sometimes quick, sometimes interrupted by sighs.
2. The pulse slow, and unequal in force and frequency.
3. Diminution of sensibility and of animal heat.
4. Alternate flushings and paleness of the countenance.
5. Drowsiness and stupor, either total or else disturbed by convulsions. Of these, the first is considered as the most certain and characteristic symptom.

The malady is said to consist essentially in an acute dropsy of the ventricles, followed by softening of the substance of the brain, and its pathology is thus explained:—The arachnoid membrane having been affected by certain causes, external or internal, from what is called its state of *primitive irritation* proceed a class of symptoms, such as head-ach, fever, vomiting, uneasiness, and cries. But, when the progress of effusion has produced compression, the pain and fever cease, and stupor ensues, with irregularity of the pulse and of respiration. The subsequent softening of the brain gives rise to what is termed the state of *consecutive irritation*, characterized by a return of fever, convulsions, asphyxia, and death.

In prosecuting his researches on the texture of the brain, M. Cruveilheir has devised a method of submitting it to the action of a jet of water; a plan, of which he proposes to make still further use in future.

The failure or insufficiency of the remedies commonly employed in this disorder, have induced the author to seek for other aid. Calomel and mercurial frictions, though not without merit, have, he thinks, been too much praised in England and Germany. He assures us that he has derived the most signal benefit from the practice of local blood-letting from the pituitary membrane, by means of a scarifying instrument which he has invented for the purpose; and he promises us the results of this practice in other disorders connected with the brain.

To the disorder which forms the principal subject of the volume the title is given of "Gastro-Intestinal Malady, or *Gastritis* or *Enteritis* of Infants, with Gelatinous Disorganization." It cannot be said that the terms marked in italics, which in their common acceptation imply *inflammation*, are happily applied to a disorder which we are expressly told is not accompanied by any; at least, upon dissection, no traces of inflammation are found. Nor does fever constitute a part of the disease: so far from it, that those cases in which it is complicated with fever appear to be the least dangerous; which is accounted for by supposing that the intestinal malady does not then prevail in so great a degree as to destroy those sympathies by which the phenomena of life and of disease are maintained.

The following is the description of the disorder:—It is ordinarily caused by premature or incautious weaning. Its principal symptoms are diarrhœa, with frequent green stools, if the disease be in the intestines; or, if it attack the stomach, vomiting of mucous or bilious matter; ardent thirst; very rapid emaciation; excessive prostration of strength; anxious countenance; a light stupor, interrupted by cries and convulsions; slow and irregular pulse, and coldness of the extremities. Its morbid result is a gelatinous disorganization, with or without perforation, of the stomach, (most frequently of its splenic extremity,) or of the small or large intestines; with thickening of their parietes, but without any trace of inflammation, or even alteration of colour.

The question is then asked, whether this disease is of recent origin? to which we should answer, certainly not; for, unless we are mistaken, its symptoms have all been enumerated by others, under the head Infantile Fever, or Remittent Fever of Infants. At the same time we are ready to admit that it has not been so fully described before, nor its morbid results clearly ascertained. The latter is easily accounted for: in the first place, examinations after death have not long been so frequent as at present; 2dly, examinations may have taken place without discovering the principal organic lesion, which, as M. Cruveilhier remarks, requires minute inspection, the viscera at a first view appearing perfectly healthy: not to mention that the ambiguous nature of the symptoms has often directed the chief attention to the head; 3dly, perforations of the stomach, when discovered, may have been attributed to the species of digestion described by Hunter, often, no doubt, erroneously; since, perhaps, the only unequivocal instances of such a process have been found in persons dying in a state of health.

The attention of our author, however, had been already called to the alteration of structure which he has described, when he had an opportunity of satisfying himself as to the nature of the disorder which leads to it, upon the occasion of its becoming epidemic at Limoges, during the latter end of the year 1819. His cases are described with extreme minuteness: of six of them the dissections are given; eleven others were attended with similar symptoms; seven cases of perforation of the stomach are collected from various authors; and, finally, several cases are added, in which this disorder appeared to exist in adults in a state of complication with other diseases. The theory by which it is attempted to account for the disorganization described above is similar to that before applied to the softening of the cerebral substance. Improper food given to infants is supposed to be the source of irritation, producing an afflux of fluids, followed by dissolution of texture, and finally by perforation of the diseased viscus. From the first state of irritation, and second of disorganization, the symptoms which have been enumerated are deduced and explained.

The only diagnosis which the author deems necessary is between this and cerebral disease. The most important diagnostic symptom is the state of sleepiness which accompanies both disorders. But in hydrocephalus it is most profound, in the other easily broken, and interrupted by cries and convulsions: in the former, in short, it is idiopathic, and merely symptomatic in the intestinal disorder. Another distinction is taken from the respiration, which is unequal, in the cerebral disease, from its commencement.

The treatment of the disorder is divided into four heads. Under that of hunger and thirst, the author professes himself, in this instance, a follower of Asclepiades, and enjoins a most severe restriction of the quantity of liquid as well as of solid food allowed to the patient; 2, he recommends a return to the breast, or at least to a milk diet; 3, he approves of warm baths; and, 4, of opiates, particularly in injections.

In the course of his remarks, M. Cruveilhier has started a couple of theories, which he has not, however, pursued far; nor do they appear, at first sight, very consistent with each other. One is, that the "gastro-intestinal malady" is connected with intermittent fevers, with which it has been sometimes complicated in the same subject, and has *once* been coexistent as an epidemic. The other theory attributes the origin of the disorder to the introduction of vaccination, and consequent suppression of the *variolous depuration.*

Of the smaller treatises affixed to this work, it is only necessary to observe that, in the first, the author states the conclusion which he has been led to form from examining the diseased lungs of some of the larger animals, that the formation of pulmonary tubercles takes place in the air-cells or bronchial vesicle. Upon this point, therefore, he agrees with the conclusion of M. Magendie, recently announced in the Journal de Physiologie. The bandage which is recommended for fractures of the clavicle is founded upon the same principles as that of Desault, the inconveniences of which it is intended to avoid. Finally, the author relates that, having accidently noticed the extreme bitterness of the capsule of the common lilac, (*syringa vulgaris,*) he has been induced to form from it a watery extract, which has produced a perfect cure in six cases of intermittent fever; a drachm of it being sufficient, administered in two days. Hence occasion is taken to express sanguine hopes of brilliant discoveries of specific remedies.

Whatever may be the fate of his theories, M. Cruveilhier undoubtedly possesses merit as a practical writer; he has observed accurately, and recorded his observations with vigour and perspicuity: but sometimes, perhaps, he raises a prejudice against himself, which detracts from the attention paid to his remarks, by an over-haste to assume the credit of novelty and discovery; claims which the reporters of the French Institute (to which he presented his manuscript,) appear to have been, on their parts, with some degree of national self-complacency, rather too ready to concede. In his own practice, he appears to display the greatest zeal and diligence, which he often endeavours to excite in others by useful exhortation. As a specimen we transcribe the following excellent precept:

"Qu'il me soit permis, à ce sujet, de faire sentir toute l'importance d'une observation attentive, minutieuse dès la première visite qu'on fait à un malade; ne disons jamais 'nous verrons demain;' tachons de donner, à l'instant même, des bases solides à notre diagnostic. Il est si facile d'embrasser une opinion erronée! et une fois notre esprit prévenu, il faut bien des jours et quelquefois bien des erreurs pour nous ramener à la vérité."—p. 125.

We shall conclude with stating our conviction that further reports of M. Cruveilhier's practice cannot but be acceptable to the medical world.

---

